# Supplementary material for: Efficient Reprogramming of Mouse Embryonic Stem Cells into Trophoblast Stem-like Cells via Lats Kinase Inhibition
Source: Biology (Basel). 2024 Jan 24;13(2):71. doi: 10.3390/biology13020071 (PMC10886645; doi:10.3390/biology13020071)
Supplement: Supplementary file 1 [file biology-13-00071-s001.zip › biology-2784198-supplementary.pdf]

Supplementary Tables S1

Antibodies used in this study.

Antigen	Manufacturer	Catalog number	Dilution
OCT4	Santa Cruz	sc-5279	1：50
GATA6	R&D Systems	AF1700	1：200
E-CAD	Abcam	ab11512	1：300
CDX2	Abcam	ab76541	1：300
KRT7	Abcam	ab181598	1：300
YAP1	My BioSource	MBS8535224	1：50
TPBPA	Abcam	ab104401	1：300
SYNA	Biorbyt	orb312961-CF405M	1：150
TFAP2C	Santa Cruz	sc-12762	1：100
GFP	R&D Systems	MAB42402-SP	1：300

Supplementary Tables S2

qRT-PCR primer was used in this study.

Target	Direction	Sequence (5'-3')
Gapdh	forward	TGTGAACGGATTTGGCCGTA
Gapdh	reverse	ACTGTGCCGTTGAATTTGCC
Cdx2	forward	CTGGACAAGGACGTGAGCAT
Cdx2	reverse	ACTGCGGAGGACTGACAAAG
Tfap2c	forward	GAGGTGCAGAATGTGGACGA
Tfap2c	reverse	CCCCAAAGGGTTCTTGGTCA
Gata3	forward	GCTCCTTGCTACTCAGGTGAT
Gata3	reverse	GGAGGGAGAGAGGAATCCGA
Tead4	forward	TTGAGCGAAGCTTCCAGGAG
Tead4	reverse	TTCCGACCATACATCTTGCCT
Gata2	forward	CGACCACACTTGTTGCACAG
Gata2	reverse	GGGTAAACAGACAGAGGCCC
Oct4	forward	GAGAAGTGGGTGGAGGAAGC
Oct4	reverse	GGAAAGGTGTCCCTGTAGCC
Sox2	forward	GATCAGCATGTACCTCCCCG
Sox2	reverse	CTGGGCCATGTGCAGTCTAC
Gata6	forward	TATTCACCAGCAGCGACTAGC
Gata6	reverse	GAGGACGAAGACGAGATGGG
PDGFRa	forward	CGCTGGAGGGTTATCGAGTC
PDGFRa	reverse	CAGGTTGGGACCGGCTTAAT
Gata4	forward	TGTGTAGCAGGCAGAAAGCA
Gata4	reverse	GGTTGCTCCAGAAATCGTGC
Eomes	forward	GGAAGTGACAGAGGACGGTG
Eomes	reverse	TTGGCGAAGGGGTTATGGTC
Elf5	forward	GTCAAGACTGTCACAGCCGA
Elf5	reverse	TTCCCATTCCAGGATGCCAC
Tfap2a	forward	TTCTGTTCAGTTCCGGGTCG
Tfap2a	reverse	CTCCCTGCTGGCAGATTCAA
Tpbpa	forward	TAGTCCCTGAAGCGCAGTTG
Tpbpa	reverse	CTGACCGGAGGCACTCATTT
Gcm1	forward	TATGTGCCCTCCCTCACGTA
Gcm1	reverse	TAAGCCCATGCATGCCATCT
Prl3d1	forward	CCCCTGTGTCATACTGCTTCC
Prl3d1	reverse	TCCTGGGAGAGCCGTCAGT
SynA	forward	CCAGCCCCACTAAGGACTTG

SynA	reverse	GACGTGGTCATCGGGGTATC
Ctsq	forward	TAGAGGCTGTCGTTGGGGTA
Ctsq	reverse	GCAATGGGGCCTTTAGTTGC
SynB	forward	GGCCCCACTCTGCATATCTC
SynB	reverse	AGCGGGTTTTTCCATTTGGC
Ascl2	forward	AGCACACCTTGACTGGTACG
Ascl2	reverse	AGTGGACGTTTGCACCTTCA
Hand1	forward	CAAGCGGAAAAGGGAGTTGC
Hand1	reverse	TCACTGGTTTAGCTCCAGCG

Supplementary Tables S3

Primers for methylated specific PCR

Target	Direction	Sequence (5'-3')
Oct4	forward	TGAGGAGTGGTTTTAGAAATAATTG
Oct4	reverse	CCACCCTCTAACCTTAACCTCTAAC
Cdx2	forward	TAAAGTGTATTTAGGTTGGAAGGAG
Cdx2	reverse	AACACAAACACCAATAACTAAAAAC
Tfap2c	forward	TGGGGAAATTTTAGTTGTTAGTTTT
Tfap2c	reverse	CCAACCAATCCCTACCTAACTC
Krt7	forward	GTTGTGGGTATTTTGGATAATTTGT
Krt7	reverse	TCTCTACAACCAAAACCTTCCTATC
Tead4	forward	TTTAAGGGATAAGGAAAGAATTGAAA
Tead4	reverse	AAAACAAAAAAAACTATTCCCTTCC

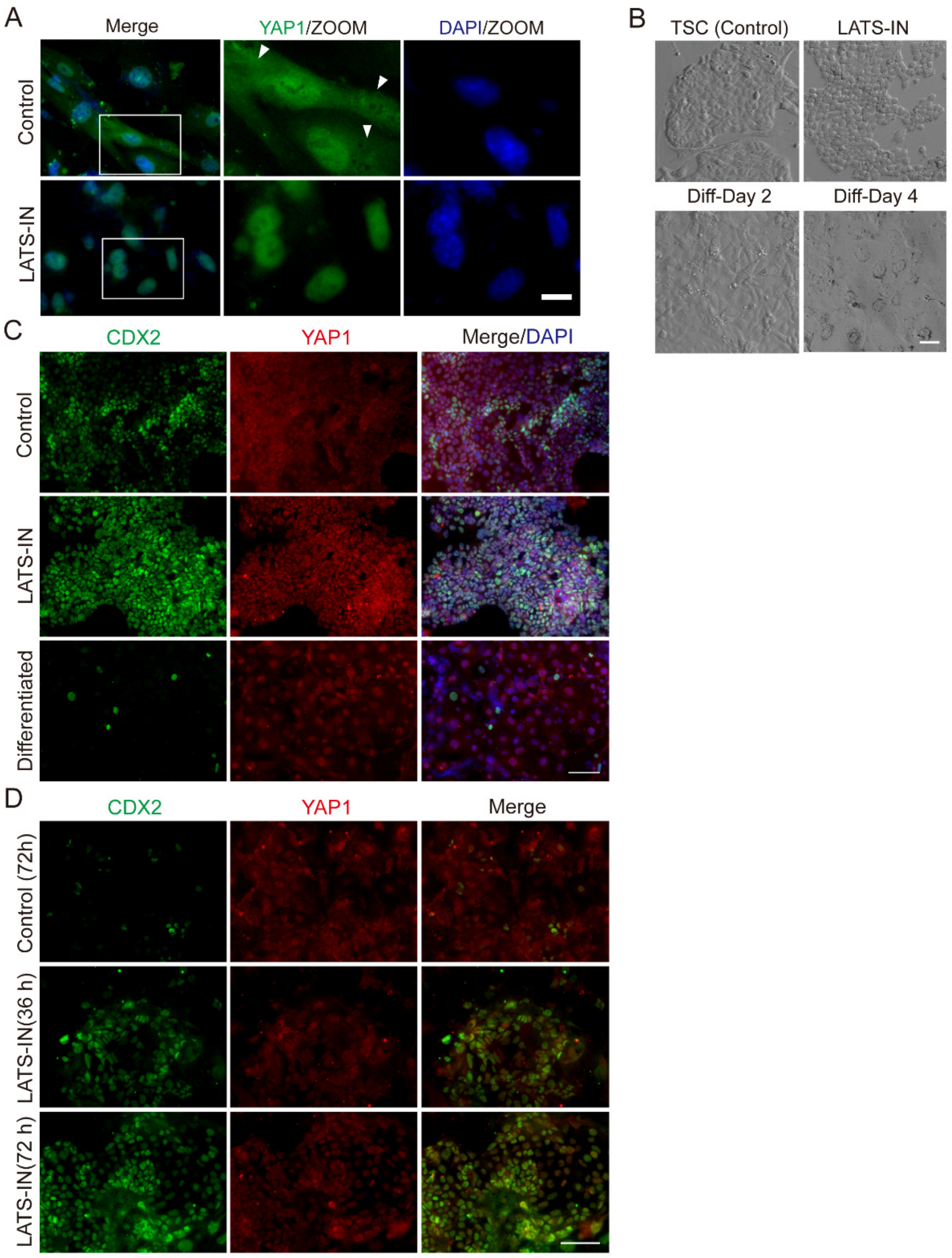

Supplementary Figure S1. The small molecule compound LATS-IN−1can promote the accumulation of YAP in the nucleus of mouse embryonic fibroblasts. **A.** Immunostaining for YAP1 of mouse embryonic fibroblasts. "LATS-IN" refers to the MEF induced with a 3μM concentration of LATS-IN−1(An inhibitor of Lats kinase) factor. Cell nuclei were stained with DAPI (blue). The white box indicates the enlarged area; white arrows indicate YAP1 accumulation in the cytoplasm **B.** Cell morphology of TSC cultured with LATS-IN−1for 2 days and differentiation for 2 and 4 days. **C.** Immunostaining for CDX2 and YAP1 of different types of trophoblast stem cells. "Control" refers to the conventional TSC culture medium; "LATS-IN" refers to the

addition of a 3μM concentration of LATS-IN−1to the TSC culture medium; “Differentiated” refers to the TSC after 3 days of random differentiation. Cell nuclei were stained with DAPI (blue). **D.** Immunostaining for CDX2 and YAP1 of differentiated TSC and differentiated TSC was treated with LATS-IN−1factor for 36h and 72h. Cell nuclei were stained with DAPI (blue). Scale bars:10μm in (A); 20μm in (B), 50μm in (C) and (D).

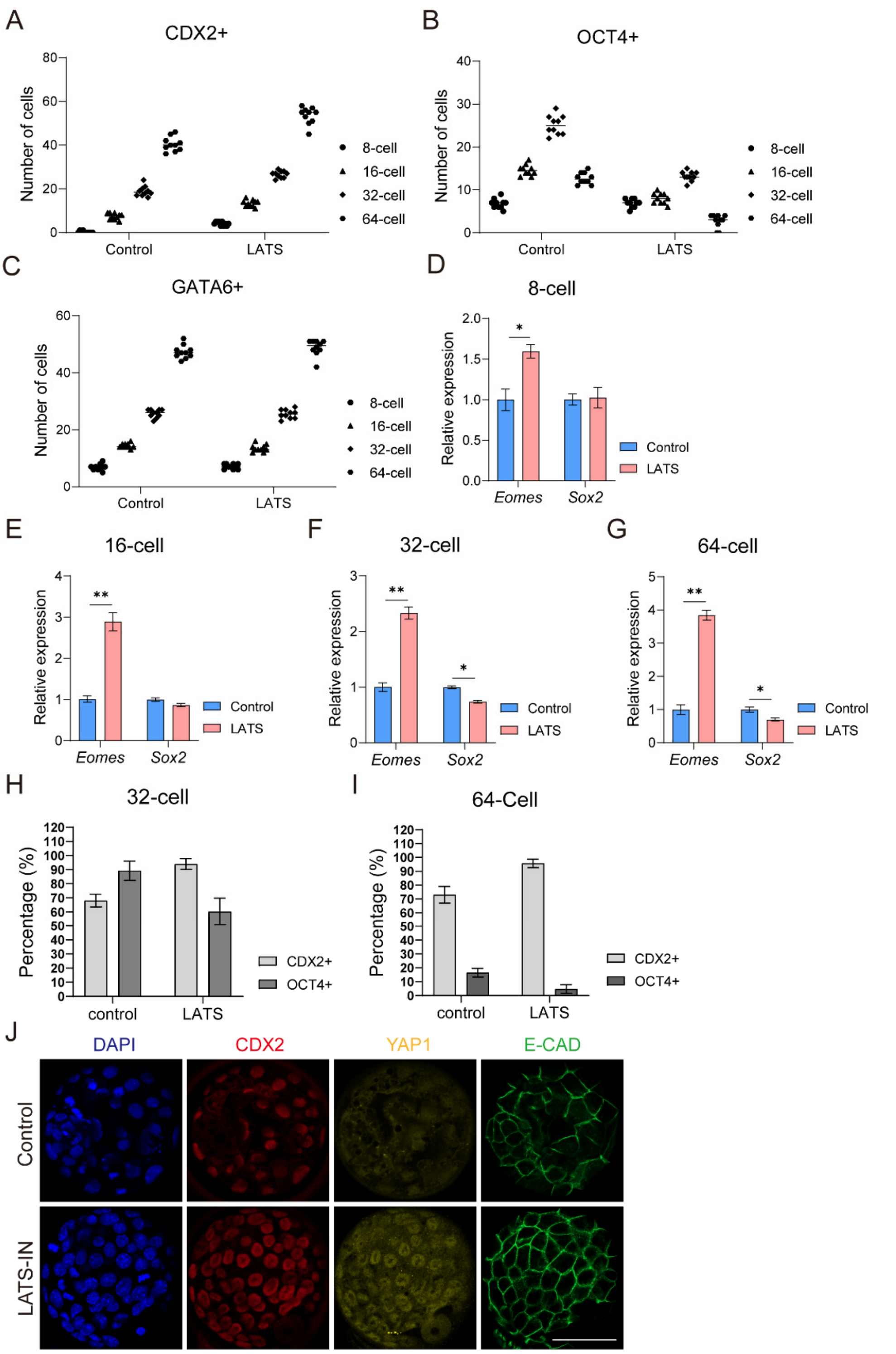

Supplementary Figure S2. Inhibition of lats kinase regulates the specialization of embryonic lineages. A-C. Comparison of the number of CDX2-positive, OCT4-positive, and GATA6-positive cells of the 8-cell stage, 16-cell stage, 32-cell stage, and 64-cell stage mouse embryos cultured with LATS-IN-1. n=10. **D-G.** Relative expression for *Eomes* and *Sox2* in the 8-cell stage, 16-cell stage, 32-cell stage, and 64-cell stage mouse embryos cultured with LATS-IN−1were detected by qRT-PCR. qRT-PCR data were normalized to Gapdh, n=3. **H.** The percentage of the number of CDX2-

positive, and OCT4-positive cells of 32-cell stage mouse embryos cultured with LATS-IN-1. n=10. **I.** The percentage of the number of CDX2-positive, and OCT4-positive cells of 64-cell stage mouse embryos cultured with LATS-IN-1. n=10. **J.** Immunostaining for E-CAD, CDX2, and YAP1 of the blastocyst (control) and LATS-IN−1induced blastocysts (LATS-IN-1). Cell nuclei were stained with DAPI (blue). The images were single focal plane, and the thickness of the optical section was 1.5μm. For each graph, the data were represented as mean ± SEM, analyzed by Student's t-test, *p < 0.05, **p < 0.01. Scale bars:50μm in (H).

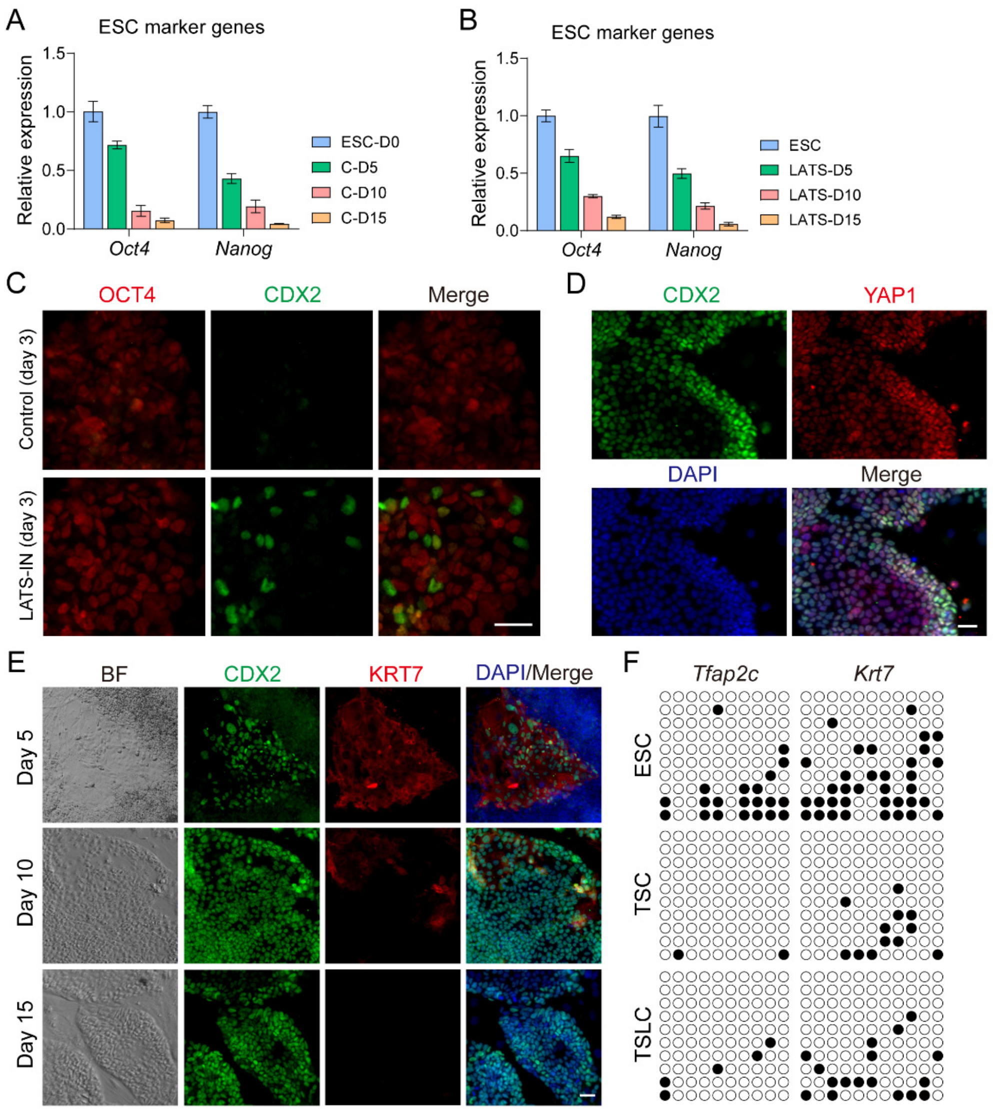

Supplementary Figure S3. Inhibition of lats kinase can reprogram ESCs into TSC-like cells.

A. Relative expression for *Oct4* and *Nanog* in ESC cultured with TSC medium for 0, 5, 10, and 15 days were detected by qRT-PCR. qRT-PCR data were normalized to Gapdh, n=3. **B.** Relative expression for *Oct4* and *Nanog* in ESC cultured with LATS-IN−1induction medium for 0, 5, 10, and 15 days were detected by qRT-PCR. qRT-PCR data were normalized to Gapdh, n=3. **C.** Immunostaining for CDX2 and OCT4 of ESC cultured with LATS-IN−1induction medium for 3 days. Cell nuclei were stained with DAPI (blue). **D.** Immunostaining for CDX2 and YAP1 of ESC cultured with LATS-IN−1induction medium for 15 days. Cell nuclei were stained with DAPI (blue). **E.** Immunostaining for CDX2 and KRT7 of ESC cultured with LATS-IN−1induction medium for 5, 10, and 15 days. Cell nuclei were stained with DAPI (blue). **F.** DNA methylation status of *Tfap2c* and *Krt7* promoter regions in ESC, TSC, and TSLC. For each gene, Bisulfite sequencing of 10 samples was performed. Open and closed circles indicated the unmethylated and methylated CpGs. Scale bars, 20μm in (C), (D), and (E).

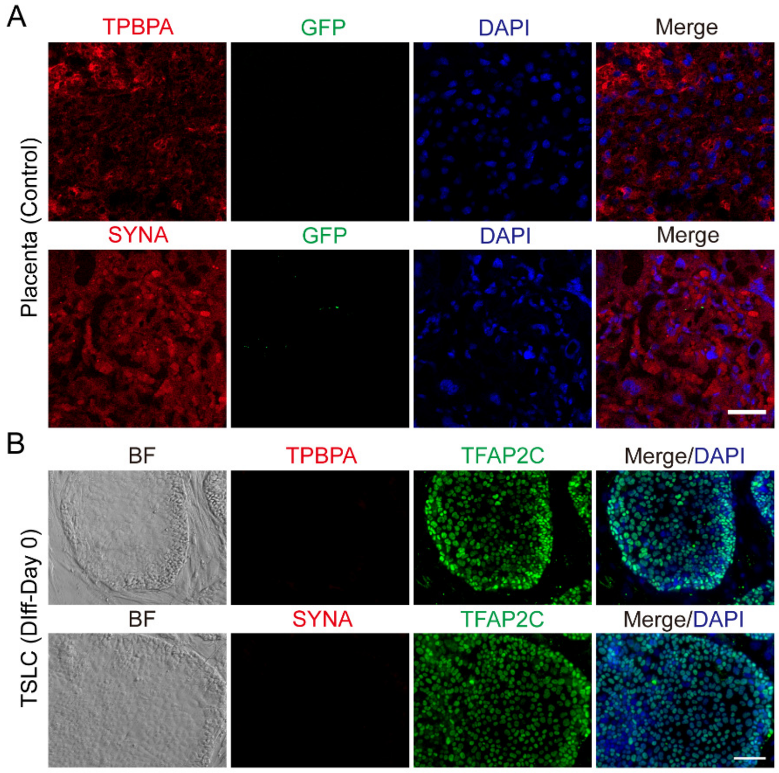

Supplementary Figure S4. TSLC has the potential to differentiate into mature trophoblast cells.

A. Immunostaining for SYNA and TPBPA of the E13.5 placenta (control). Cell nuclei were stained with DAPI (blue). **B.** Expression of TPBPA, SYNA, and TFAP2C of undifferentiated TSLCs were detected by immunofluorescence. Cell nuclei were stained with DAPI (blue). Scale bars: 20μm in (A), 50μm in (B).
